# Differentiation of hepatic alveolar echinococcosis with a hemangioma-like pattern compared to typical liver hemangioma using contrast-enhanced ultrasound: a pilot study

**DOI:** 10.1007/s15010-022-01866-z

**Published:** 2022-07-01

**Authors:** Jana Philipp, Julian Schmidberger, Patrycja Schlingeloff, Wolfgang Kratzer

**Affiliations:** Department of Internal Medicine I, Ulm University Hospital, Albert-Einstein-Allee 23, 89081 Ulm, Germany

**Keywords:** Alveolar echinococcosis, *Echinococcus multilocularis*, Hepatic hemangioma, Ultrasonography, Contrast-enhanced ultrasound, EMUC-US

## Abstract

**Purpose:**

*Echinococcus multilocularis* infects humans as a false intermediate host, primarily with intrahepatic manifestation. Incorrect diagnostic interpretation of these liver tumors, especially the hemangioma-like pattern, can lead to progressive disease. The aim of the study was to investigate the differentiation of typical hemangioma and a hemangioma-like pattern of *E. multilocularis* using contrast-enhanced ultrasound (CEUS).

**Methods:**

This prospective clinical pilot study comprised patients with hemangioma (*n* = 14) and patients with alveolar echinococcosis (AE) and hemangioma-like pattern (*n* = 7). Inclusion criteria were the detection of a liver lesion according to a hemangioma-like pattern on *E. multilocularis* Ulm classification—ultrasound (EMUC-US) and “confirmed” or “probable” AE according to WHO case definition. The comparison group had hepatic hemangioma with typical B-scan sonographic morphology. All participants underwent conventional and contrast-enhanced ultrasonography.

**Results:**

The patient group comprised five men (71.4%) and two women (28.6%) with a mean average age of 64.1 ± 11.2 years. The patient group with hemangioma comprised nine female subjects (64.3%) and five male subjects (35.7%) with a mean average age of 56.1 ± 12.0 years. Early arterial bulbous ring enhancement (*p* < 0.0001) and iris diaphragm phenomenon could only be visualized in the patients with hemangioma (*p* < 0.0001). Furthermore, the patients with hemangioma exhibited hyperenhancement in the late phase (*p* = 0.0003). In contrast, the patients exhibited typical early arterial rim enhancement (*p* < 0.0001) and, in the portal venous and late phase, complete or incomplete non-enhancement (black hole sign; *p* = 0.0004).

**Conslusion:**

The behavior of hemangioma-like AE lesions and typical liver hemangiomas is significantly different on CEUS. AE should be considered as a possible differential diagnosis, especially in high-endemic areas.

## Introduction

Fox tapeworm disease, alveolar echinococcosis (AE) is a zoonosis caused by the larval stage of the cestode *Echinococcus multilocularis*, for which humans may act as a false intermediate host. The worm eggs are ingested accidentally, penetrate the small intestinal wall and travel to organs via the blood or lymphatic vessels. In a majority of cases, AE primarily affects the liver and forms infiltrative alveolar space-occupying lesions, similar to a malignant tumor [1, 2].

Increasing prevalence and incidence, and spread beyond the classic endemic areas, have been reported worldwide [3]. After infection with *E. multilocularis*, fox tapeworm disease is characterized by an asymptomatic progressive course. Five to 15 years can pass before a diagnosis of AE is made [1, 2]. Due to the potentially lethal course of AE, therapy must be initiated after diagnosis [1]. First-choice therapy is radical surgical resection [4]. In the case of inoperability, life-long oral antihelmintic therapy with benzimidazole (BZM) is usually required [5]. However, initial data indicate that negative serology for echinococcus and lack of ^[18F]^FDG uptake in ^18^FDG positron emission tomography (PET) suggests that discontinuation of mebendazole therapy is feasible [6–8].

The diagnosis of AE is based on the combination of imaging, clinical and serological findings [4]. As an inexpensive, radiation-free and readily available technique, ultrasound is the method of choice for detecting the disease [9]. The heterogeneous sonomorphology of hepatic AE, especially the metastasis- and hemangioma-like pattern, poses a major challenge in clinical practice [10].

**Fig. 1:**
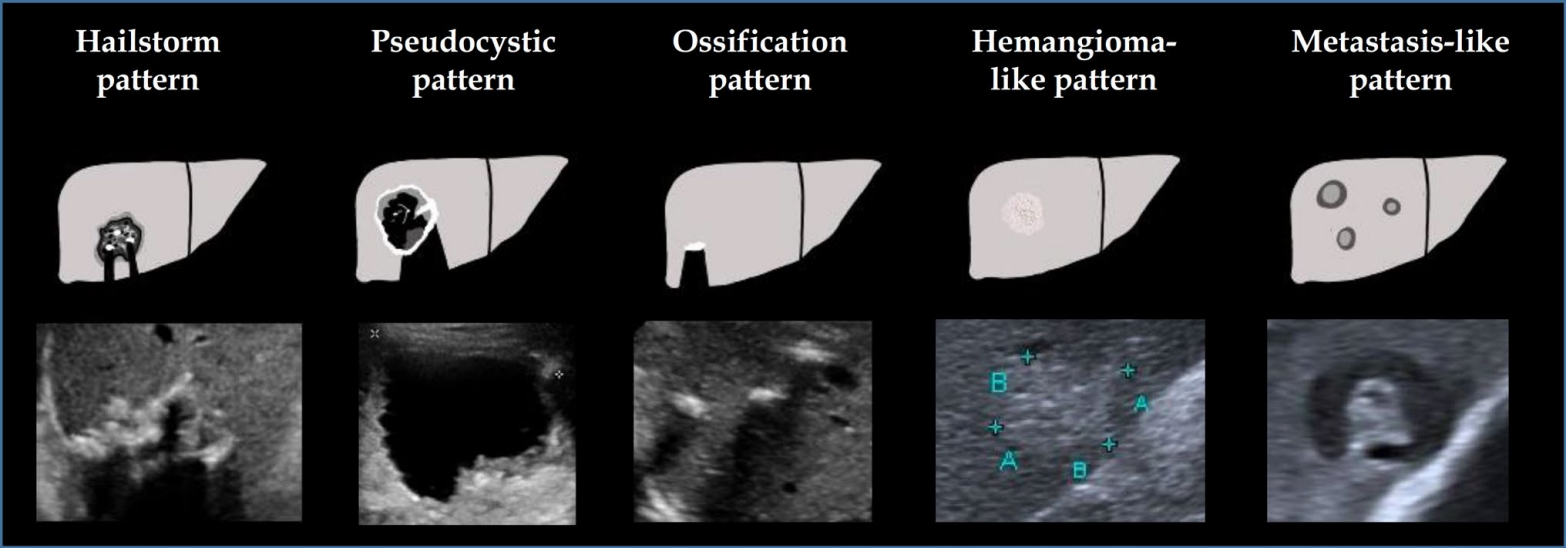
*Echinococcus multilocularis* Ulm classification—ultrasound (EMUC-US) [10]

The *E. multilocularis* Ulm classification—ultrasound (EMUC-US) was the first to systematically describe the different morphologies [10] (Fig. 1). A total of five following different patterns are distinguished: hailstorm, pseudocystic, hemangioma-like, metastasis-like and ossification [10]. CT and MRI are additional methods that can be used in the diagnosis of hepatic AE [11–13]. However, PET-CT and PET-MRI are the only methods used to assess the parasitic activity of AE lesions [14, 15]. More than 30% of patients are misdiagnosed during the diagnostic workup [16]. This can lead to delayed therapy, progression of the disease and an increase in complication-rich and inoperable disease courses [5, 16]. Differentiation of typical hemangiomas and a hemangioma-like pattern according to EMUC-US is not possible with B-scan ultrasonography alone.

Contrast-enhanced ultrasound (CEUS) has emerged in recent years as another diagnostic technique in AE. However, the studies that have investigated the use of CEUS in hepatic AE have been almost exclusively retrospective, in rodents, or not used a classification system [11, 17–21]. No prospective studies investigating the hemangioma-like pattern in AE are available. Hemangiomas are one of the most common tumors in the liver, along with cysts [22]. Contrast-enhanced sonography is the method of choice in the workup of typical hemangiomas [23, 24].

Based on the knowledge that the use of contrast-enhanced sonography already allows more accurate characterization of AE lesions, this prospective study is the first to investigate the differences in the contrast response of patients with hemangioma-like pattern classified by EMUC-US and subjects with typical liver hemangiomas. The aim of this study was to investigate the extent to which “typical true hemangiomas” indistinguishable sonomorphologically on B-scan differ from hemangioma-like lesions in hepatic AE on CEUS.

## Methods

### Ethics statement

This study was approved by the local ethics committee and conducted in accordance with the Declaration of Helsinki (ref. no. 17/21 and 23/20). All data were analyzed pseudo-anonymously.

### Study collective

**Fig. 2:**
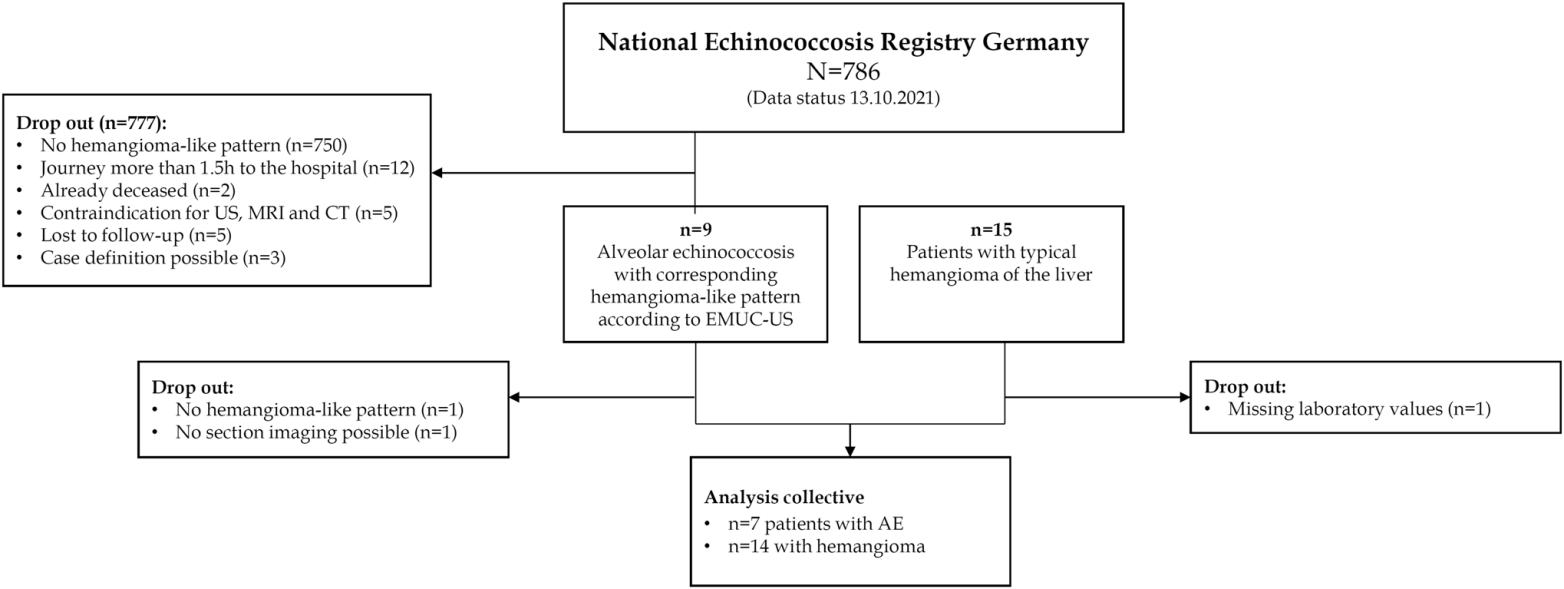
Flowchart illustrating the number of patients with hemangioma-like pattern in AE and patients with typical hemangioma included in this study starting with the alveolar echinococcosis (AE) database in Germany

**Fig. 3:**
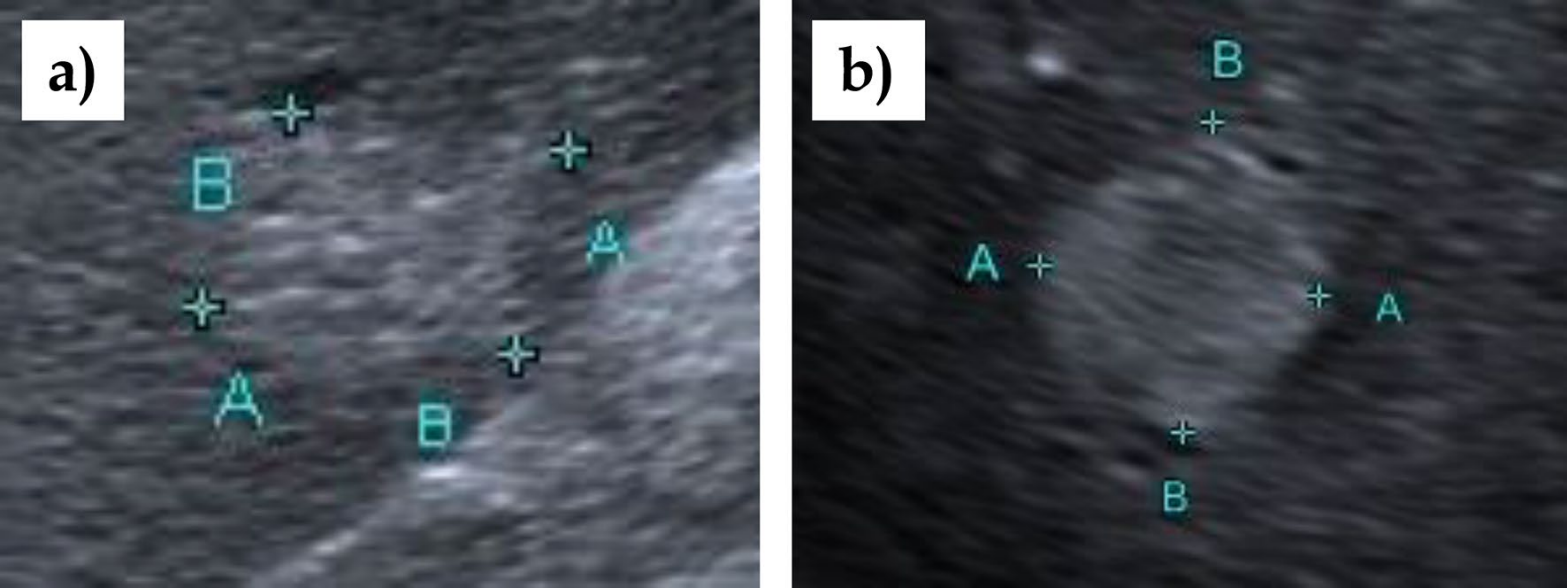
**a** Hemangioma-like pattern on B-scan according to EMUC-US; **b** Liver hemangioma on B-scan

The prospective clinical study was conducted from March 2021 to June 2021 (Fig. 2). The study population consisted of a patient group with hemangioma-like pattern in alveolar echinococcosis and a comparison group with typical hemangioma. Patients with AE were recruited from the National Echinococcosis Registry [25]. Patients were contacted by telephone or mail as part of the prospective study and were invited to participate in the study. Inclusion criteria were the presence of a liver lesion with hemangioma-like pattern classified by EMUC-US and confirmed or probable AE disease according to WHO case definition “confirmed” or “probable” [4] (Fig. 3a). For the comparison group, patients with simple liver hemangioma on central ultrasound were recruited during the same time period. During the study period, 15 consecutive patients who presented for evaluation of hemangiomas in our ultrasound department were recruited and asked to participate in the study. Inclusion in the patient group with hemangioma required liver hemangioma with typical B-scan sonographic morphology (round to oval shape, homogeneously echo-rich, sharp border) (Fig. 3b).

### Medical history

All patients were asked about their current health status before enrollment in the study and were appropriately examined during sonography if they had symptoms. A comprehensive vegetative and anthropometric history (age, height, weight) was obtained from all patients and subjects before conventional ultrasound and CEUS were performed.

### Ultrasound examinations

Ultrasound examinations were performed using Canon/Toshiba Aplio i800 (convex probe, C6-1MHz) and Toshiba Aplio 500 (convex probe, C5-2MHz; Toshiba i8CX1, Canon Medical Systems Corporation) ultrasound scanners. In the B-scan, the liver was scanned in tissue harmonic imaging (THI) mode, and all fox tapeworm lesions and hemangiomas were measured at their greatest extent in at least two planes, assigned to a liver segment (according to Couinaud), precisely described morphologically and examined for blood flow using standard Doppler techniques (color-coded/power (CPA)/advanced dynamic imaging (ADF)/superb microvascular imaging (SMI) Doppler). If there was evidence of cholestasis or tenderness, this was also noted on the examination form. The lesion with the largest diameter was the reference lesion.

### Contrast-enhanced ultrasound

Following the screening of the liver in the B-scan, CEUS was performed according to the “Guidelines and Good Clinical Practice Recommendations for Contrast Enhanced Ultrasound in the Liver—Update 2020” of the World Federation for Ultrasound in Medicine and Biology [26]. For a better overview of the liver parenchyma, twin mode was activated and the device standardized to 8.00–22.00 frames per second (fps), a low mechanical index (MI) of 0.06–0.09, a gain of 84.00 on average (range 71.00–88.00) and a dynamic range of 60.00–75.00. After selecting an optimal transducer position for the examination with the best possible visualization of the reference lesion, 1.2–1.8 ml of SonoVue^®^ (Bracco Medical Imaging Deutschland GmbH, Konstanz, Germany) was administered intravenously via an indwelling vein cannula within 2 s, followed by 10 ml of 0.9% NaCl solution. Two subjects received 1.2–1.8 ml SonoVue^®^ again for better visualization of the reference lesion. “Contrast arrival time” was defined as the time until the contrast agent flared up, marking the beginning of the arterial phase, which changes to the portal venous phase after 30 s due to increasing uptake of contrast medium by the liver parenchyma. The portal venous phase is followed by the late phase after 120 s. The reference lesion was observed by CEUS for a total of 4 min. Compared to the surrounding liver parenchyma, the contrast agent behavior of the reference lesion was semi-quantitatively described as hyper-, iso-, hypo-, or non-enhancement in the arterial, portal venous and late phases by an experienced ultrasound investigator. In hepatic hemangiomas, typical behavior on CEUS has been defined as early arterial bulbous ring enhancement followed by complete or incomplete iris diaphragm phenomenon [23, 27].

### Statistical analysis

Statistical analyses were performed using SAS version 9.4 (SAS Institute Inc., Cary, NC, USA). First, the frequencies, means, medians and location and dispersion measures were calculated. Normal distribution was assessed using the Shapiro–Wilk test. For a comparison of interval-scaled variables, the non-parametric Mann–Whitney *U* test was used. Pearson’s χ^2^ test and Fisher’s exact test were used to determine possible relationships and differences in frequency distributions between dichotomous variables. *p*-values < 0.05 (*α* = 0.05) were considered significant with a 5% probability of error.

## Results

### Study collective

The patient group (*n* = 7) included five men (71.4%) and two women (28.6%). At the time of the study, the mean average age was 64.1 ± 11.2 years and the mean BMI was 24.9 ± 3.9 kg/m^2^. Five patients (71.4%) were classified as “probable” and two (28.6%) as “confirmed” according to the WHO case definition for AE. The mean duration of disease in AE patients was 47.3 ± 28.5 months. At diagnosis, three patients (42.9%) reported symptoms, including upper abdominal tenderness and fullness. Four patients (57.1%) were asymptomatic and the diagnosis of AE was made as an incidental finding. All patients were treated with oral antihelmintic therapy. The patient group with hemangioma (*n* = 14) included nine female subjects (64.3%) and five male subjects (35.7%). The mean average age was 56.1 ± 12.0 years and the mean BMI was 25.1 ± 2.8 kg/m^2^. There were no significant differences in gender, age, or BMI between the patients with hemangioma and patient with hemangioma-like pattern in AE (Table 1).

**Table 1:** Overview of patients with hemangioma-like pattern in AE and patients with typical hemangioma

	AE group		Typical hemangioma	
	*n* (%)	Mean ± SD (median)	*n* (%)	Mean ± SD (median)
Quantity	7 (100.0)		14 (100.0)	
Gender				
Female	2 (28.6)		9 (64.3)	
Male	5 (71.4)		5 (35.7)	
Age (years)		64.1 ± 11.2 (63.0)		56.1 ± 12.0 (57.5)
BMI (kg/m^2^)		24.9 ± 3.9 (25.3)		25.1 ± 2.8 (25.1)
Disease duration (months)		47.3 ± 28.5 (36.0)		
Case definition				
Confirmed	2 (28.6)			
Probable	5 (71.4)			
Localization of the reference lesion				
Right hepatic	2 (28.6)		9 (64.3)	
Left hepatic	2 (28.6)		5 (35.7)	
Bihepatic	3 (42.9)		0 (0.0)	
Number of lesions				
1	4 (57.1)		12 (85.7)	
2	1 (14.3)		1 (7.1)	
3	2 (28.6)		1 (7.1)	
Lesion size (mm)		61.9 ± 30.0 (61.0)		21.2 ± 12.8 (22.0)
Therapy for AE				
Initial albendazole	7 (100.0)			
Therapy changed to mebendazole	1 (14.9)			
Surgical therapy	0 (0.0)			
No therapy (rejection by patients)	0 (0.0)			
Duration BMZ-Therapy (months)		43.6 ± 28.0 (30.0)		
PNM-Staging				
P3N0M0	2 (28.6)			
P4N0M0	5 (71.4)			

*AE* Alveolar echinococcosis, *SD* Standard deviation, *BMI* Body mass index

### Lesion size, localization and morphology on the B-scan

A total of 12 AE lesions and 17 hemangiomas were detected on B-scan. Four patients with AE (57.1%) and 12 patients with hemangioma (85.7%) presented with one solitary liver lesion, one patient with AE (14.3%) and one patient with hemangioma (7.1%) with two, and two patients with AE (28.6%) and one patient with hemangioma (7.1%, 1/14) with three hepatic space-occupying lesions. The mean lesion size was 61.9 ± 30.0 mm for the AE patients and 21.2 ± 12.8 mm in patients with hemangioma. The difference in the mean reference lesion size between the patients with hemangioma and patients with AE was significant (*p* = 0.0019).

The reference lesion was located left hepatic in two patients with AE (28.6%) and five patients with hemangioma (35.7%) and right hepatic in two patients with AE (28.6%) and nine with hemangioma (64.3%). Bihepatic involvement was observed in three patients (42.9%).

Significant differences were found in the morphology of the liver lesions in regard to the shape of the space-occupying lesions; 6 (85.7%) of the AE lesions had a polycyclic shape, whereas all 14 (100.0%) hemangiomas had round-oval presentation (*p* = 0.00012). Furthermore, we found significant differences in the lesions in terms of demarcation from the liver parenchyma; all 7 (100.0%) AE lesions had blurred demarcation, whereas 11 (78.6%) hemangiomas were sharply demarcated from the liver tissue and the remaining 3 (21.4%) had slightly blurred demarcation (*p* < 0.0001). Regarding homogeneity, significant differences were identified between the space-occupying lesions (100.0%) inhomogeneous presentation of AE lesions vs. 85.7% [12/14] homogeneous hemangiomas and 14.3% [2/14] inhomogeneous hemangiomas (*p* = 0.00031). All hemangiomas presented without perfusion using standard Doppler techniques (Table 2).

**Table 2:** Differences in behavior between patients with simple hemangiomas and patients with alveolar echinococcosis and hemangioma-like pattern

	AE-patients (*n* = 7)	Typical hemangioma (*n* = 14)	*p*-value
Arterial phase			< 0.0001
Rim enhancement			
No	0 (0.0%)	14 (100.0%)	
Yes	7 (100.0%)	0 (0.0%)	
Arterial phase-Bulbous ring enhancement			< 0.0001
No	7 (100.0%)	0 (0.0%)	
Yes	0 (0.0%)	14 (100.0%)	
Portal venous phase Central non-enhancement (black-hole sign)			0.0004
No	0 (0.0%)	11 (78.6%)	
Complete	5 (71.4%)	0 (0.0%)	
Incomplete	2 (28.6%)	3 (21.4%)	
Portal venous phase-Iris phenomenon			< 0.0001
No	7 (100.0%)	0 (0.0%)	
Incomplete	0 (0.0%)	3 (21.4%)	
Complete	0 (0.0%)	11 (78.6%)	
Late phase-Central non-enhancement (black-hole sign)			0.0004
No	0 (0.0%)	11 (78.6%)	
Complete	5 (71.4%)	0 (0.0%)	
Incomplete	2 (28.6%)	3 (21.4%)	
Late phase-Hyperenhancement			0.0003
No	7 (100.0%)	2 (14.3%)	
Yes	0 (0.0%)	12 (85.7%)	
Late phase-Iso-enhancement			0.5333
No	7 (100.0%)	12 (85.7%)	
Yes	0 (0.0%)	2 (14.3%)	
Late phase-Hypo-enhancement			0.1000
No	5 (71.4%)	14 (100.0%)	
Yes	2 (28.6%)	0 (0.0%)	

Data are frequency (%)

### Behavior of lesions in CEUS

A mean contrast arrival time of 14.7 ± 4.9 s was determined for patients and 11.6 ± 2.1 s for patients with hemangioma.

### AE lesions

**Fig. 4:**
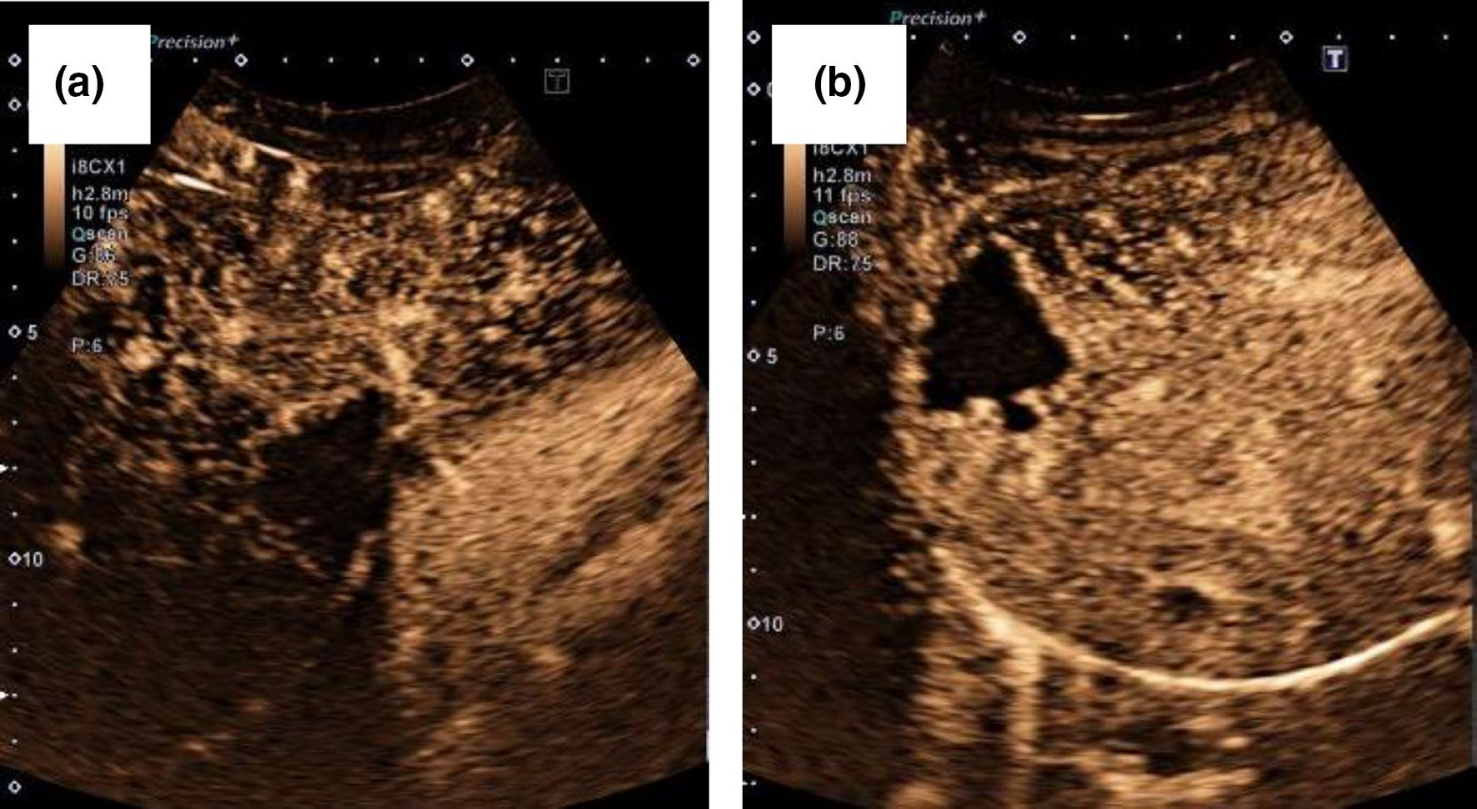
**a** Typical early arterial rim enhancement in a histologically confirmed AE lesion; **b** typical hemangioma in the arterial phase with suggested bulbous ring enhancement and beginning iris diaphragm phenomenon

**Fig. 5:**
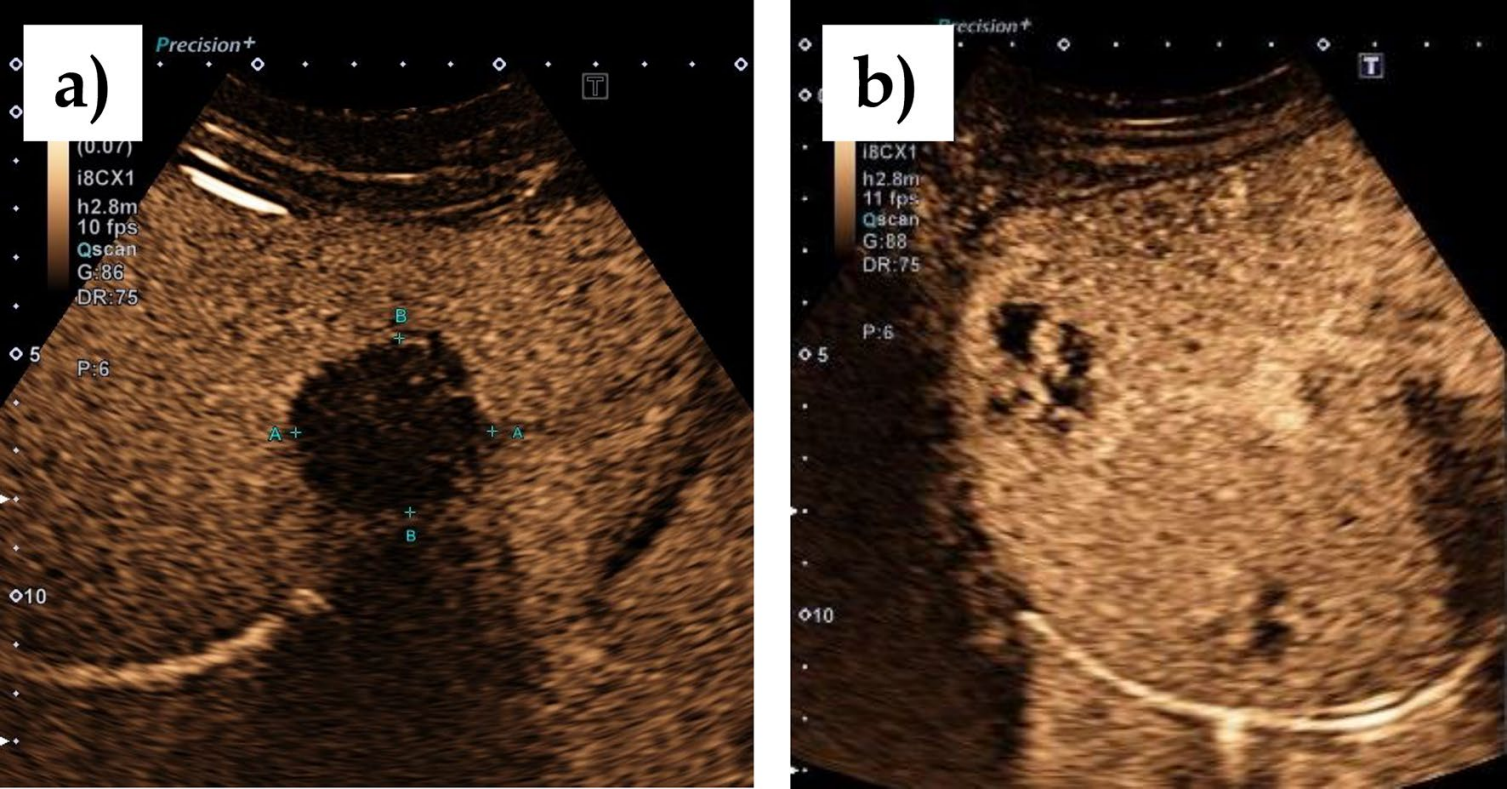
**a** In the portal venous phase the AE lesion shows no contrast enhancement in the sense of a black hole phenomenon; **b** Increasing contrast uptake in the sense of an iris diaphragm phenomenon in the portal venous phase in a typical hemangioma

**Fig. 6:**
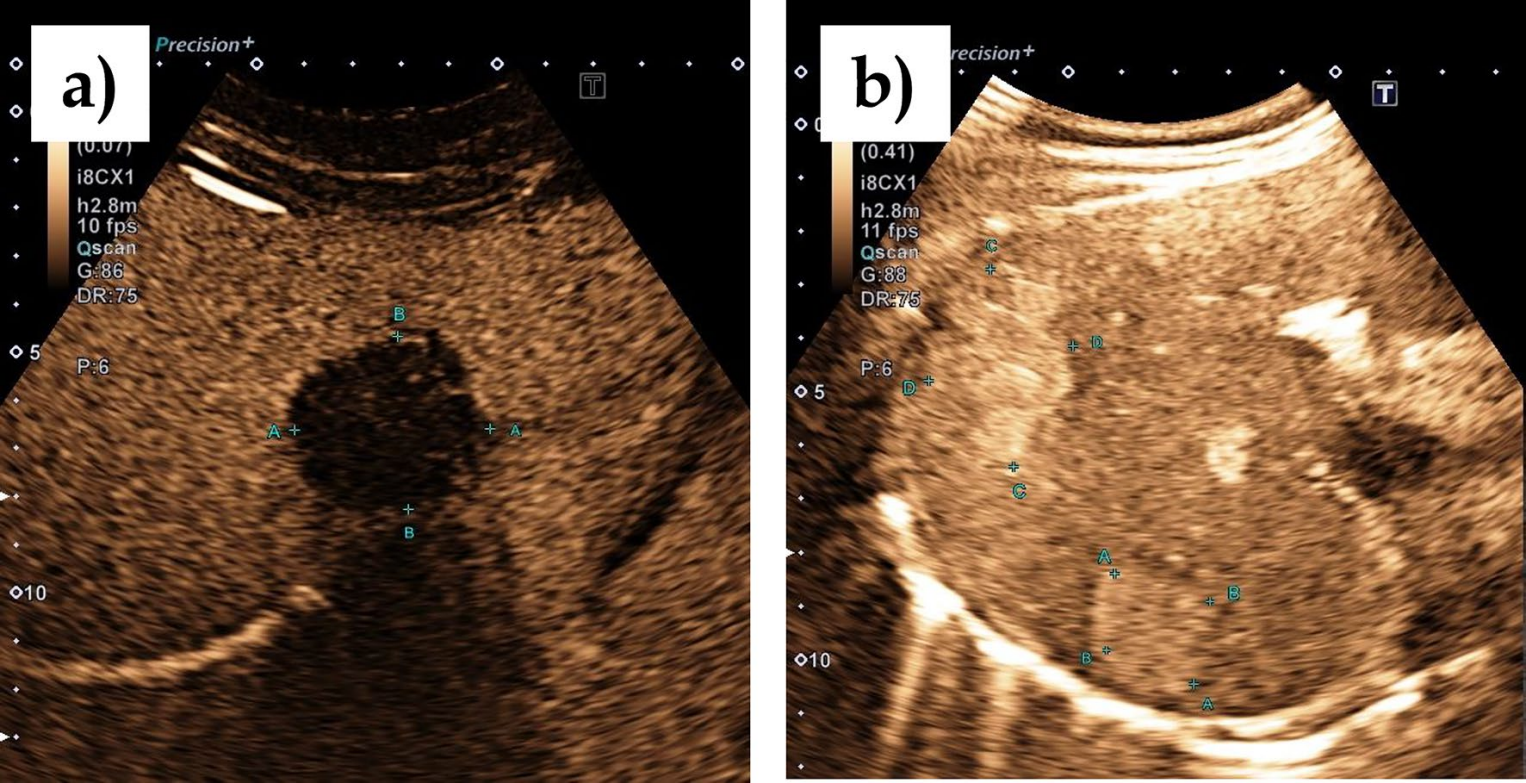
**a** In the late phase, the AE lesion continues to show no contrast uptake. Further demonstration of the existing black hole phenomenon; **b** complete contrast uptake in the sense of a complete iris diaphragm phenomenon and echo-rich visualization of the hemangioma compared to the adjacent liver parenchyma

All AE lesions presented early arterial rim enhancement in the arterial phase (*p* < 0.0001) (Fig. 4a). In one patient (14.3%), this presented as atypical and fan-shaped. An iris phenomenon was not observed in any AE lesion during the portal venous phase (*p* < 0.0001) (Fig. 5a). All AE lesions presented with central non-enhancement (complete or incomplete) in the portal venous and late phases (*p* = 0.0004) (Fig. 6a). No significant difference was found for the irregular central hypo-enhanced internal echoes observed in two (28.6%) AE patients (*p* = 0.1000) (Table 2).

### Hemangiomas

In contrast, bulbous ring enhancement was observed in all hemangiomas in the arterial phase (*p* < 0.0001) (Fig. 4b). In the portal venous phase, 3 hemangiomas (21.4%) presented incomplete and 11 (78.6%) complete iris phenomenon (*p* < 0.0001) (Fig. 5b). Of the 14 hemangiomas, 12 presented with hyper-enhancement (*p* = 0.0003) and 2 with iso-enhancement (*p* = 0.5333) in the late phase (Fig. 6b, Table 2).

## Discussion

This is the first prospective clinical study to evaluate AE patients with a hemangioma-like pattern classified by EMUC-US in contrast-enhanced sonography and compare them to patients with typical hepatic hemangioma. The results of this study support the hypothesis that typical hemangiomas and AE space-occupying hemangioma-like lesions differ significantly on CEUS.

Multiple prior studies also observed early arterial rim enhancement and black hole sign in fox tapeworm lesions [11, 17–21]. However, these studies were mostly performed retrospectively or in rodents and did not classify the AE lesions according to EMUC-US.

The hemangioma-like pattern of AE and its difficult differential diagnosis from atypical hemangioma on B-scan ultrasonography was first described by Bresson-Hadni et al. in 2006 [28]. Consistent with our study results, Cai et al. postulated that CEUS, in contrast to B-scan ultrasound, provides better visualization of morphological and vascular structures in the differential diagnosis of AE. In their study, five AE lesions could be diagnosed only by CEUS. On B-scan ultrasonography, the echo-rich space-occupying lesions were misinterpreted as typical hemangiomas [17]. Comparative studies with other diagnostic modalities and liver lesions of other entities are necessary to establish CEUS as a diagnostic tool in AE with atypical sonomorphology. However, such comparative studies are currently lacking. In addition to EMUC-US, classification systems currently exist for MRI and CT [12, 13]. In 2015, Azizi et al. correlated MR tomographic AE lesions classified by Kodama with ^[18F]^FDG uptake on PET-CT and demonstrated increased PET activity for type I–III microcysts [29]. Given the potential for AE to change its morphological presentation during the course of disease, the retrospective design of this investigation with sometimes months-long intervals between diagnostic modalities represents a major shortcoming [30]. Our study results support the need to perform a one-time CEUS to exclude fox tapeworm disease in the case of an incidentally first diagnosed echo-rich mass of the liver (suspected typical hemangioma) in high-endemic areas and the concomitant presence of risk factors for AE [31–33].

### Limitations

The small number of included patients with AE with a hemangioma-like pattern must be considered a limitation. Furthermore, the patients with hemangioma consisted of nine women and only five men. However, a possible different contrast behavior of hemangiomas between women and men has not been described in the literature so far. Due to the rarity of the disease in Germany, further multicenter studies are necessary to confirm the results.

## Conclusions

Our work shows that hemangioma-like AE lesions classified by EMUC-US differ from typical liver hemangiomas on CEUS. In the presence of early arterial rim enhancement and black hole sign, the diagnosis of AE should be considered. When a hemangioma-like liver lesion is first diagnosed, CEUS of the liver should be performed in high-endemic areas to rule out hepatic AE.

## Data Availability

The datasets used and analyzed during the current study are available from the corresponding author on reasonable request.

## Declarations

### STROBE statement

The authors have read the STROBE Statement checklist of items, and the manuscript was prepared and revised accordingly.
